# Synthesis and Study of Palladium Mono- and Bimetallic (with Ag and Pt) Nanoparticles in Catalytic and Membrane Hydrogen Processes

**DOI:** 10.3390/nano12234178

**Published:** 2022-11-24

**Authors:** Polina Pushankina, Mikhail Baryshev, Iliya Petriev

**Affiliations:** 1 Department of Physics, Kuban State University, 350040 Krasnodar, Russia; 2 Laboratory of Problems of Stable Isotope Spreading in Living Systems, Southern Scientific Centre of the RAS, 344006 Rostov-on-Don, Russia

**Keywords:** nanoparticles, nanostructured surface, catalytic activity, methanol oxidation reaction, palladium-containing membranes, hydrogen permeability

## Abstract

A controlled strategy for the electrochemical synthesis of mono- and bimetallic nanoparticles with a unique and complex morphology has been developed. The investigation of the effect of changing the surfactant concentration and current density regulating the medium pH has revealed the fundamental patterns of nanoparticle growth. The developed method has allowed to synthesis of nanoparticles with a controlled pentabranched structure for the monometallic palladium as well as for favorable combinations of metals—Pd-Ag and Pd-Pt. The obtained nanoparticles were investigated in alkaline methanol oxidation. The results demonstrated quite high catalytic activity up to 83.51 mA cm^−2^ and long-term stability, which are caused by the increase in electrochemically active surface area by increasing the active center’s number. This was made possible due to the creation of unusual nanoparticle morphology, namely the presence of high-energy high-index facets. The developed nanoparticles were also studied as a modifying coating for hydrogen-permeable membranes in the processes of hydrogen transport. The membranes coated with the nanoparticles demonstrated sufficiently high hydrogen flux up to 11.33 mmol s^−1^ m^−2^ and high H_2_/N_2_ selectivity up to 2254. Such results can be explained by the obvious acceleration of surface processes through the application of the developed nanoparticles. The novel synthesis strategy can potentially be extended to other metal nanoparticle systems. Thus it can be an effective way to solve relevant problems of design of controlled synthetic methods allowing the nanoparticle morphology tuning according to the required functional properties.

## 1. Introduction

Nowadays, the nanotechnology field has significantly strengthened its position in many branches of human activity [1,2,3,4,5,6]. The design, development, and study of nanostructures attract the great attention of scientists all around the world because of the wide range of nanoparticle applications. Such studies are significantly dependent on the search for new stable and reproducible methods for obtaining nanoparticles with required parameters. For example, T. Takai et al. [7] have investigated the nanoparticles microwave synthesis implementation by the addition of surfactant. This method has allowed stabilization of synthesis via the synergistic effect of two-stage microwave treatment and a surfactant presence. In turn, S.S. Chan et al. [8] carried out microalgae-mediated nanoparticle synthesis. The method has proved to be quite stable and reduced the number of harsh chemicals involved in the synthesis. However, electrolytic deposition is the most interesting and promising method from the whole variety of known synthesis strategies [9,10,11,12]. The essence of the electrolytic deposition method is to create or modify particles on the electrode surface in a reaction cell containing a growth solution, where a potential or current causes the particle’s growth. The fundamental processes underlying the deposition reaction have been studied from different sides, such as kinetic, mechanistic, thermodynamic, and elastic continuum, each of which covers different aspects of the problem. However, a core understanding of the deposition process can be gained from the resulting coating morphology analysis. The morphology of a coating formed in a solid matrix is determined by many factors, among which the most significant consist of interfacial energy, strain energy, and deposition temperature. Many articles [13,14,15,16,17] confirm the presence of structural sensitivity in a variety of nanomaterials. This means that the size, shape, spatial distribution, and composition of nanoparticles, especially noble metal ones, are key parameters for tuning their activity and reactivity [18,19,20,21,22].

Nanoparticles are most widely used in medicine [23,24], spectroscopy [25,26], catalysis [27,28], and energy [29,30]. Catalysis and energy fields are of main interest in this paper, since the development of advanced catalysts based on metal nanoparticles with improved activity, selectivity and stability is an important and relevant task [31,32,33]. One of the most interesting and effective ways to improve the catalytic properties of nanomaterials is to manipulate the shape of the nanoparticles. Such an approach makes it possible to obtain nanoparticles with the required surface structure, i.e., with a well-defined arrangement of surface atoms. Nevertheless, the resulting particles are far from model; their structure is complex and includes various kinds of defects (for example, irregular twin boundaries, corners, vacancies) and surface domains of various geometries and sizes, i.e., the surface structure determines the catalytic activity of the particles in the chosen reaction to a greater extent than even the shape [34]. Therefore, a significant aspect that modern nanoscience is striving for, is the controlled synthesis of functional nanoparticles.

The composition plays a great role in the properties tuning of synthesized nanomaterials along with the shape and structure. Palladium is well-known as the most widespread and promising metal for electrocatalysis and membrane applications [35,36,37]. Pd is used in a huge variety of configurations affecting the physical and chemical properties of material during synthesis. For example, E. Garnier et al. [38] have synthesized and investigated cubic, octahedral, and rhombododecahedral Pd nanoparticles with predominant surface structures {100}, {111}, and {110}, respectively. The electrochemical response was directly determined by the surface structure of the particle according to the research. However, monometallic systems do not always have the required electrocatalytic activity. Therefore, a synergistic effect between different elements leads to catalytic properties optimization. In this regard, H. Lv et al. [39] have developed and investigated a three-component Pd-B-P catalytic system in electrochemical oxygen reduction. The developed catalyst exhibited catalytic performance that exceeded previously reported values for current Pd-based catalyst analogs. Therefore, the aim of this study is to develop a stable and reproducible electrochemical synthesis strategy that allows the manipulation of the shape and structure of nanoparticles based on Pd, Pd-Ag, and Pd-Pt, as well as investigating the obtained particles as catalysts in alkaline methanol electrooxidation. The articles on the electrochemical synthesis of particles nowadays are mostly focused on the modification of pre-synthesized pseudospherical particles or completely do not consider the shape and structure of the formed particles [40]. However, this research pays special attention to obtaining the one-stage seeds and their growth into nanoparticles with the required shape and structure. This widens the range of sizes and shapes of the resulting particles and allows direct manipulation of the synthesis components, such as the halide ions and surfactant concentration as well as the medium pH.

## 2. Materials and Methods

### 2.1. Nanoparticles Synthesis and Morphology Investigation

Synthesis of classical palladium black nanoparticles was carried out by the method described in [35,41].

The electrolytic deposition of monometallic palladium nanoparticles was carried out on a P-40X potentiostat-galvanostat in the galvanostatic mode. The metal film (palladium or palladium-silver) was washed in 96% ethanol and degreased by boiling for 30 min in 6 M NaOH (98%) solution before the deposition. After such preparation, the palladium-containing film was transferred to an electrolytic cell, where it was polarized anodically in 0.1 M hydrochloric acid (38%) and cathodically in 0.05 M sulfuric acid (95%) at a current density of 10–20 mA cm^−2^. Next, the cell was filled with a growth solution containing C_16_H_36_BrN (99%) surfactant along with 2% H_2_PdCl_4_ (99.9%). Very low current densities up to 0.003 mA cm^−2^ were set for a short period of 30–60 s to carry out the process of nucleation on the film surface, after which the current density increased to 0.25–0.3 mA cm^−2^, providing further particle growth for 3.5–10 min. At the end of the deposition process, the film was washed with bidistillate.

The synthesis of palladium-silver nanoparticles was carried out also in the galvanostatic mode. The palladium-containing films were washed in 96% ethanol, degreased by boiling for 30 min in 6 M NaOH (98%) solution, and anodically and cathodically polarized in 0.1 M HCl (38%) and 0.05 M H_2_SO_4_ (95%), respectively, at a current density of 10–20 mA cm^−2^. The growth solution contained 2% H_2_PdCl_4_ (99.9%), 0.005 M AgNO_3_ (99.5%), and C_16_H_36_BrN (99%). For deposition, a stepwise current mode was set like for monometallic nanoparticles starting from low values of current density up to 0.003 mA cm^−2^ for 30–60 s and instantly raised to 0.25–0.3 mA cm^−2^ for 3.5–10 min. The film was washed with bidistillate after deposition.

The synthesis of palladium-platinum nanoparticles was carried out in the direct current mode by galvanic exchange. Likewise, the films were washed in 96% ethanol, degreased by boiling for 30 min in 6 M NaOH (98%) solution, and anodically and cathodically polarized in 0.1 M HCl (38%) and 0.05 M H_2_SO_4_ (95%), respectively, at a current density of 10–20 mA cm^−2^. Next, the cell was filled with a growth solution of 2% H_2_PdCl_4_ (99.9%) and C_16_H_36_BrN (99%). During the deposition process, a low current density was initially set up to 0.003 mA cm^−2^ for 30–60 s, after which it was raised to 0.25–0.3 mA cm^−2^ for a longer period of time for 3.5–10 min. After deposition, the film was washed with bidistillate. All reagents were supplied by Sigma-Aldrich.

The morphology of the obtained nanoparticle samples was studied using a JEOL JSM-7500F scanning electron microscope.

### 2.2. Nanoparticles Investigation in Catalytic and Membrane Applications

The catalytic activity of the developed nanoparticles was studied by cyclic voltammetry in the alkaline methanol oxidation in the potential range from −0.9–0.5 V at the scanning speed of 50 mV s^−1^ at room temperature. The growth solution was 1 M sodium hydroxide with 0.5 M methanol. The measurements were carried out on a P-40X potentiostat-galvanostat in a three-electrode cell, which consisted of a working electrode (samples of palladium films covering developed catalysts), a counter electrode (platinum electrode) and a reference electrode (silver chloride glass electrode), relative to which the potentials were reported.

The long-term stability of the developed nanoparticles was studied by chronoamperometry in the reaction of alkaline oxidation of methanol at a constant potential of −0.3 V in the time range of 0–2400 s.

The study of the processes of hydrogen transport of the developed membrane samples (Pd/Pd, Pd-Pt/Pd, and Pd-Ag/Pd) was carried out on a hydrogen permeability unit, according to the method described in [41].

## 3. Results and Discussion

### 3.1. Development and Control of Metal Nanoparticles Synthetic Methods

The controlled electrochemical methods for the Pd-based monometallic nanoparticles synthesis have been developed. Synthesis was carried out by electrolytic deposition at direct current and by chemical deposition in a colloidal solution. Such an approach has allowed us to carry out a comparative analysis of both methods, which, in turn, made it possible to choose the most suitable and to obtain a clear understanding of the particle synthesis fundamental patterns. The electrochemical approach seems to be the most promising and simple synthesis method. The electrochemical synthesis uniqueness lies in the additional synthesis control (current/potential), which can be used as a control tool for the shape and structure of the resulting particles. A comparison of chemical and electrochemical methods was carried out through a series of experiments with the main parameters for controlling the morphology of the synthesis of nanoparticles—the concentration of the surfactant and pH control in the growth solution.

**Figure 1:**
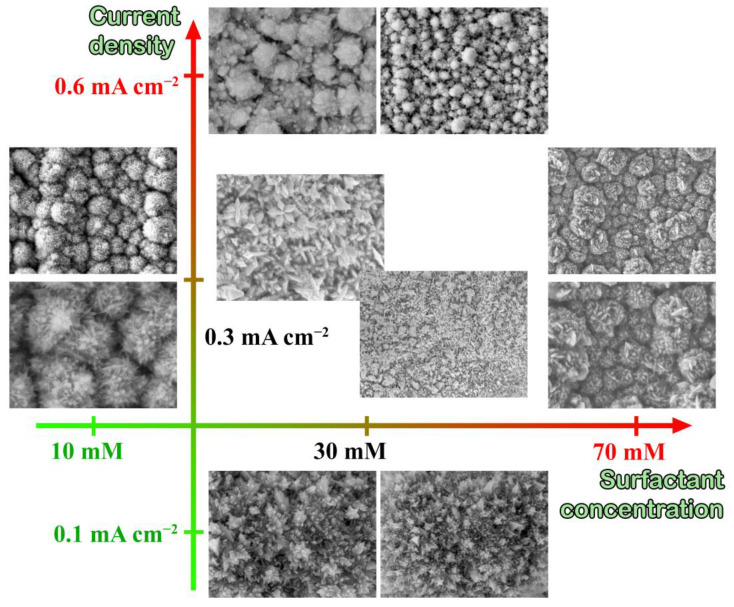
SEM images of the monometallic palladium nanoparticles shaping depending on the surfactant concentration and the current density in the process of electrochemical synthesis.

The obtained palladium nanoparticles morphology dependency on surfactant concentration was studied in the first series of experiments. Various surfactant concentrations with other parameters kept at optimal conditions in colloidal and electrochemical synthesis turned out to give quite similar results. The particles formed a more rounded shape as the concentration of the surfactant (tetrabutylammonium bromide) increased which may be due to the increase in the surface coverage of its molecules. Probably, a reaction rate decreased in a colloidal solution and a cell potential increased in the electrochemical system caused this effect. The growing particles tend to form more energetically favorable spherical configurations under such conditions. On the contrary, a surfactant concentration decrease leads to the formation of particles with a more clearly defined structure. Figure 1 shows SEM images of a series of particles synthesized with a surfactant concentration from 10 to 70 mM. The obtained results allowed us to conclude that the most optimal concentration for both types of synthesis is 30 mM.

The second series of experiments was aimed at investigating the effect of changing the medium (pH of the growth solution) on the resulting nanoparticle morphology. M.L. Personick et al. [42] studied the effect of medium pH changing under the conditions of colloidal nanoparticle synthesis. The presence of a reducing agent (Personick et al. used ascorbic acid) is obligatory in colloidal synthesis. It controls the palladium ions reduction rate in the growth solution. In turn, changing the medium pH, which is carried out in this article by changing the nitric acid concentration, is the main way to control the reduction rate during nanoparticle synthesis. The absence of concentrations and ultra-low concentrations of nitric acid (from 10 µL to 50 µL) led to high pH values, therefore, accelerated the kinetics of the reducing agent which resulted in the formation of the single-crystal structures without special morphology. On the contrary, a nitric acid concentration increase entailed a medium pH decrease and led to a reaction rate decrease. Such slow kinetics allowed the particles to line up in well-defined shapes and form complex morphologies. However, polydisperse inhomogeneous particles of different sizes were observed under conditions of a significantly increased nitric acid concentration (up to 50 µL) and, consequently, a critically decreased pH. Thus, the nitric acid concentration synthesis of 30 µL was considered as most optimal for chemical colloidal.

The current applied to the cell plays the role of the reducing agent in the electrochemical synthesis. Therefore, the medium pH has a weaker effect on the particle growth rate than in the colloidal synthesis without a chemical reducing agent in the growth solution. Such a control allows for the ability to direct the growth of specific facets of the particle surface and to shape it. In one-stage electrochemical synthesis, a small current of 0.003 mA cm^−2^ was initially applied to the working cell (to the electrodes) and it initiated the particles nucleation on the electrode surface. The current was increased to 0.1–0.6 mA cm^−2^ causing further growth of nanoparticles after that. Such current increase reproduces the effects of changing the medium pH in colloidal synthesis. The following dependence was observed in electrochemical synthesis: a significant current increase led to a deposition rate increase and, consequently, to particle thickening on the electrode surface. A significant current decrease resulted in low-index particles with no particular morphology. According to carried out experiments, 0.25–0.3 mA cm^−2^ was chosen as the optimal current value in the process of particle growth.

The carried-out experiments allow speaking about the uniqueness and simplicity of the use of electrochemical synthesis. This method makes it possible to successfully synthesize nanoparticles with the required morphology due to their strong adsorption on the electrode surface during growth as well as due to the chemical-reducing agent absence that directly affects the medium pH in colloidal synthesis.

### 3.2. Morphology and Shaping Characteristics of the Synthesized Mono- and Bimetallic Nanoparticles Based on Pd, Pd-Ag, Pd-Pt

**Figure 2:**
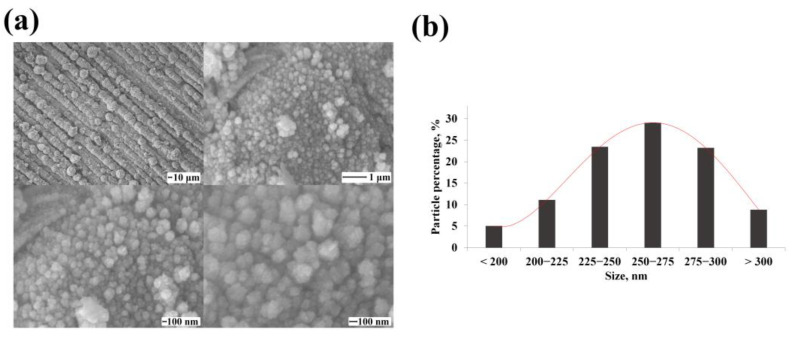
(**a**) SEM images of synthesized classical palladium black nanoparticles at various magnifications; (**b**) Size distribution diagram of synthesized classical palladium black nanoparticles.

The classical methods of electrolytic deposition synthesis allow obtaining spherical particles of an energetically favorable configuration. Figure 2a shows SEM images of classical particles synthesized by electrochemical deposition. On average, 52.5% of the synthesized nanoparticles were the size of 225–275 nm according to the particle size distribution diagram (Figure 2b).

The experiments on the influence of the synthesis medium conditions (changes in the surfactant concentration and pH) have made it possible to expand the obtainable range of nanoparticle shapes and morphologies. Based on these data, the most optimal proportions of the components and synthesis parameters have been chosen, which make it possible to obtain monometallic palladium particles with a nonclassical pentagonal morphology. The developed strategy of controlled synthesis has been applied to bimetallic systems—Pd-Ag and Pd-Pt.

**Figure 3:**
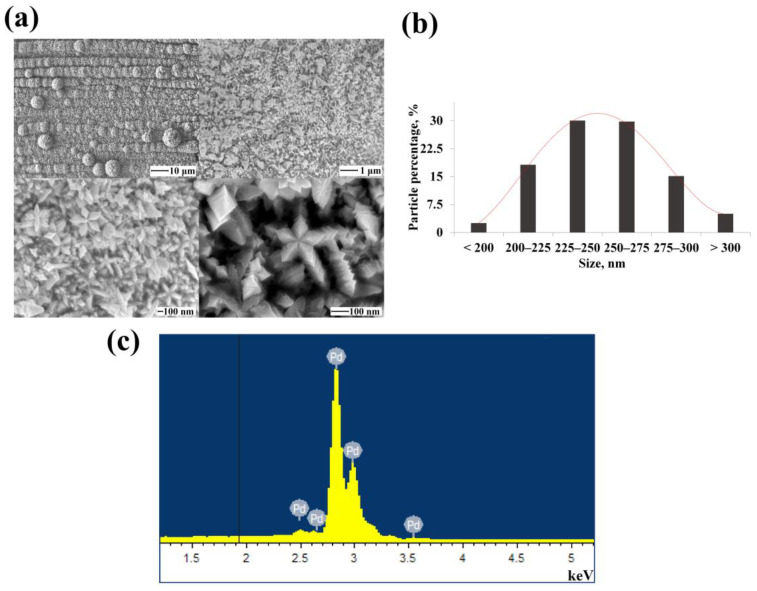
(**a**) SEM images of synthesized monometallic palladium nanoparticles at various magnifications; (**b**) Size distribution diagram of synthesized monometallic palladium nanoparticles; (**c**) EDS spectra of the elemental composition of synthesized monometallic palladium nanoparticles.

Figure 3a shows SEM images of electrochemically synthesized monometallic palladium nanoparticles. These had a pentabranched structure with high-index facets and defects in the twins structure. On average, 59.8% of the synthesized nanoparticles were of the size 225–275 nm according to the particle size distribution diagram (Figure 3b). The results of the EDS analysis presented in Figure 3c showed 99.92% palladium content and few impurities in the synthesized functional layer.

**Figure 4:**
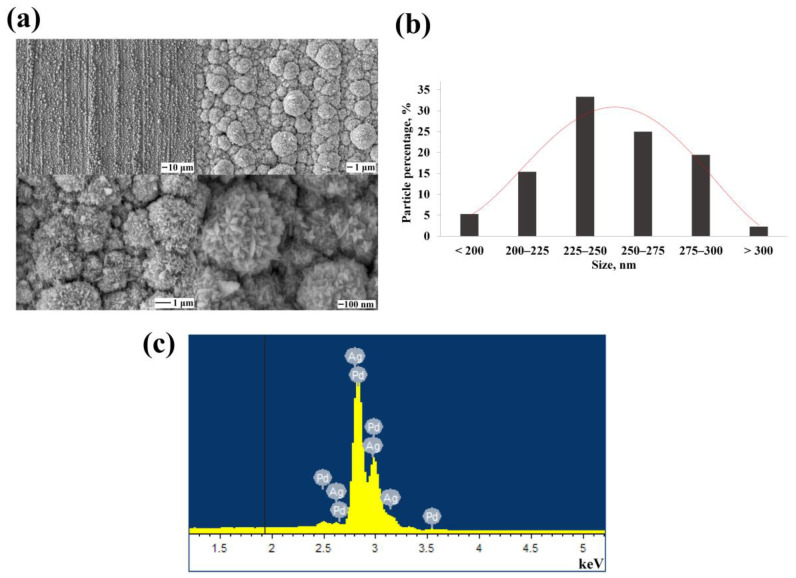
(**a**) SEM images of the synthesized bimetallic Pd-Ag nanoparticles at various magnifications; (**b**) Size distribution diagram of the synthesized bimetallic Pd-Ag nanoparticles; (**c**) EDS spectra of the elemental composition of synthesized bimetallic Pd-Ag nanoparticles.

The pentagonally structured monometallic particles obtaining technique was applied to alloys of palladium with silver and platinum. Figure 4a shows SEM images of electrochemically synthesized bimetallic Pd-Ag nanoparticles. This palladium-silver alloy system is highly preferable, since both metals have fcc lattice, close electronegativity on the Pauling scale with a difference of 0.27, and their radii differ by only 5% with respect to palladium [43]. In addition, this bimetallic system follows Vegard’s law with a slight deviation: the lattice parameter of the palladium-silver alloy continuously increases with the growth of silver in solid solution along a curve that deviates from the law towards compression up to 0.001 Å [44]. Such a Pd-Ag system is of great interest for application in catalysis due to its special properties, such as increased selectivity in hydrogenation reactions [45]. It is known that the catalytic activity of Pd-Ag nanoparticles strongly depends on the structural characteristics. M. Guo et al. [46] studied the structural characteristics using an advanced genetic algorithm systematically followed by molecular static modeling. According to the results, silver atoms tend to occupy surface positions in the formation of a nanoparticle, while palladium atoms prefer to occupy central positions due to the lower surface energy of silver atoms (1320) compared to palladium atoms’ surface energy (2043). However, metals form miscible solid solutions in experiments because of the influence of experimental conditions. As well as monometallic particles, the nanoparticles synthesized in this experiment formed a shape resembling a five-pointed star, formed due to high-index facets and twinning defects. The obtained configuration confirms the twinning ability not only of noble monometals but also of their combinations. On average, 58.3% of the synthesized bimetallic Pd-Ag nanoparticles were of the size 225–275 nm according to the particle size distribution diagram (Figure 4b). The results of the EDS analysis presented in Figure 4c showed 88.53 % palladium and 11.47 % silver content in the synthesized functional layer.

**Figure 5:**
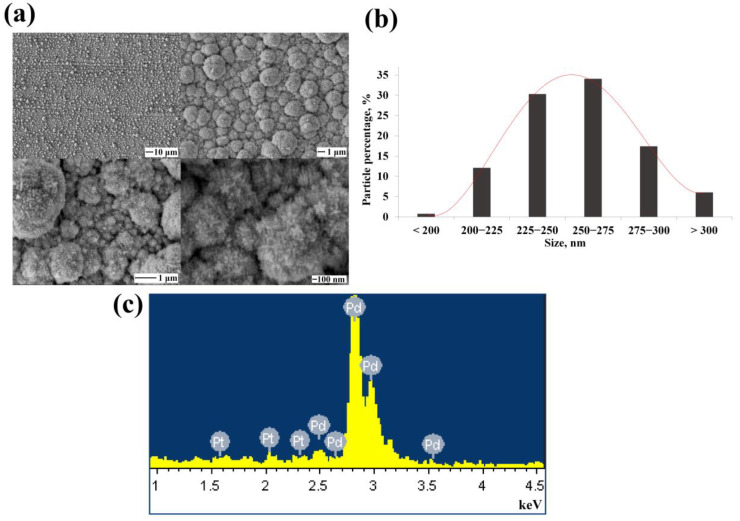
(**a**) SEM images of the synthesized bimetallic Pd-Pt nanoparticles at various magnifications; (**b**) Size distribution diagram of synthesized bimetallic Pd-Pt nanoparticles; (**c**) EDS spectra of the elemental composition of synthesized bimetallic Pd- Pt nanoparticles.

The developed synthesis strategy was also proven on the Pd-Pt bimetallic system. Figure 5a shows SEM images of electrochemically synthesized bimetallic Pd-Pt nanoparticles. The selected metals are quite similar in characteristics and application. Both metals have fcc lattice, a difference in radii of only 1.5%, close standard reduction potentials, even closer than for palladium with silver, and electronegativity on the Pauling scale with a difference of 0.08, which allow for expecting negligible charge transfer between the two elements. The Pd-Pt system, like Pd-Ag, has a negative deviation from Vegard’s law, which confirms the stability of a disordered solid solution over the entire temperature range beyond crystallization. L.O. Paz-Borbon et al. [47] confirmed through density functional (DF) theory calculations the preference for the arrangement of platinum atoms in central positions with palladium atoms on the surface of Pd-Pt alloy nanostructures due to the higher cohesive energy and higher surface energy of platinum relative to palladium at almost similar atomic radii. In this experiment, the aim was also to synthesize nanoparticles with pentabranched star shapes. The obtaining of a similar shape and morphology of mono- and bimetallic nanoparticles is crucial to understanding and evaluating the effects of secondary metals in catalytic and membrane studies most accurately. On average, 64.4% of the synthesized bimetallic Pd-Pt nanoparticles were of the size 225–275 nm according to the particle size distribution diagram (Figure 5b). The results of the EDS analysis presented in Figure 4c showed 93.72% palladium and 6.28% platinum content in the synthesized functional layer.

The electrochemical synthesis method proved to be a rather successful reproducible strategy for the synthesis of nanoparticles with a controlled preferred architecture. This method is suitable for the manufacturing of particles of binary alloys palladium with silver or platinum as well as for monometallic palladium particles. The adaptation and application of the developed synthesis technique of certain shaped metal alloy nanoparticles is significantly important in the development of the most interesting and in-demand electrocatalytic systems.

### 3.3. Electrochemical Study of Synthesized Mono- and Bimetallic Nanoparticles Based on Pd, Pd-Ag, Pd-Pt

**Figure 6:**
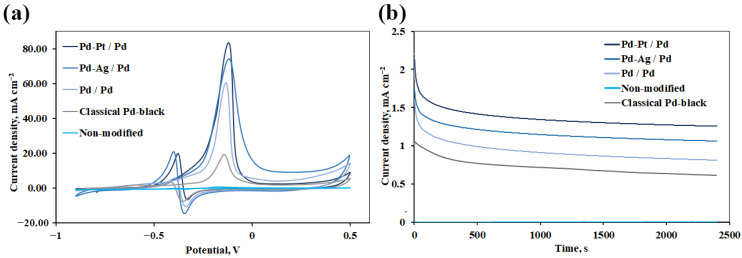
(**a**) Cyclic voltammograms of the developed mono- and bimetallic nanoparticles as catalysts in the alkaline oxidation of methanol (1 M NaOH + 0.5 M CH_3_OH); (**b**) Chronoamperometric curves of the developed mono- and bimetallic nanoparticles as catalysts in the alkaline oxidation of methanol (1 M NaOH + 0.5 M CH_3_OH) at a fixed potential of −0.3 V.

The catalytic activity of electrochemically synthesized palladium-based mono- and bimetallic nanoparticles has been studied by cyclic voltammetry in the methanol oxidation in an alkaline medium. Cyclic voltammetry plots were obtained in a 1.0 M NaOH solution with the addition of 0.5 M methanol at a potential sweep rate of 50 mV s^−1^ in the voltage range from −0.9 V to 0.5 V. All modified electrodes showed similar trends in the current density peaks in the forward and reverse sweeps of potential, thus all electrodes catalyzed methanol oxidation in the potential range above -0.25 V. There are two irreversible current peaks in the anode zone during forward and reverse sweep for each electrode in Figure 6a. The first peak in the forward sweep can be associated with the oxidation of freshly chemisorbed particles getting from the methanol adsorption. The second peak in the reverse sweep may be due to the removal of incompletely oxidized carbonaceous particles formed during forward scanning. It should be noted that the peak current density value in a forward sweep is directly proportional to the electrocatalytic activity of palladium-based methanol oxidation catalysts.

The catalysts synthesized on the surface of the Pd electrode had a sufficiently high activity and demonstrated the following peak current densities: for Pd-Pt/Pd—up to 83.51 mA cm^−2^, for Pd-Ag/Pd—up to 73.62 mA cm^−2^ and for Pd/Pd—up to 60.39 mA cm^−2^. The classical Pd_black_/Pd particles had a significantly lower activity of 18.94 mA cm^−2^. Such an increase in the catalytic activity of bimetallic catalysts most likely may be due to the synergistic effect of the secondary metal (silver or platinum), which also inhibits the poisoning of catalyst active sites more efficiently than the monometal [48,49]. In addition, the initial potential of the Pd-Pt/Pd (up to −0.26 V) catalyst is more negative than for Pd-Ag/Pd (up to −0.25 V), Pd/Pd (up to −0.24 V) and Pd_black_/Pd (−0.23 V). A more negative shift for the initial potential indicates that methanol can be easily oxidized and CO intermediates forming during methanol oxidation can be easily removed [50]. An initial potential with a more positive shift indicates less adsorption of methanol on the catalyst surface due to highly adsorbed carbonaceous species, which in turn leads to a decrease in the current response at a certain potential.

The resistance of the developed mono- and bimetallic catalysts to the accumulation of intermediate forms of CO during the methanol oxidation was evaluated using the peak current densities ratio obtained from the ratio of anodic peaks in forward and reverse scan (I_f_/I_b_). A low I_f_/I_b_ value indicated poor methanol electrooxidation, suggesting less alcohol adsorption on the catalyst surface due to excessive accumulation of carbonaceous intermediates. On the contrary, high ratio values indicated that the catalyst tends to be more resistant to intermediate forms of CO and thus facilitates the oxidation of methanol to form carbon dioxide. The developed catalysts demonstrated sufficiently high stability up to 4.35 for Pd-Pt/Pd, up to 3.57 for Pd-Ag/Pd, up to 5.96 for Pd/Pd, up to 6.39 for Pd_black_/Pd.

Long-term stability is another important factor for catalysts. It is known that some carbonaceous particles form in redox reactions on the surface of the nanoparticles, easily inactivating the catalyst. [51]. Therefore, chronoamperometric measurements were carried out to evaluate the electrochemical stability of the developed mono- and bimetallic catalysts. Figure 6b shows that the polarization of developed Pd-Pt/Pd, Pd-Ag/Pd, and Pd/Pd catalysts decayed rapidly during the first 300 s due to chemisorbed carbonaceous particles, after which the hyperbolic decay slowly reached a pseudo-stationary state. The Pd-Pt/Pd catalyst retained 75% of its original current density after the first 300 s, outperforming the Pd-Ag/Pd (up to 72%), Pd/Pd (up to 59%) and Pd_black_/Pd (up to 80.5%) catalysts. The preservation of a greater percentage of the original current density, and therefore a greater value of the stabilized current density, indicates a lower rate of catalyst poisoning during methanol electrooxidation reactions. Hence, the developed Pd-Pt/Pd, Pd-Ag/Pd, and Pd/Pd catalysts are capable of maintaining sufficiently high current density and low breakdown rate during the entire scan time, demonstrating excellent electrocatalytic activity and methanol oxidation stability.

Thus, the developed mono-(Pd/Pd) and bi-metal (Pd-Pt/Pd and Pd-Ag/Pd) catalysts have demonstrated excellent electrocatalytic activity and long-term stability with respect to methanol electrooxidation. Such results may probably be due to the increased electrochemically active surface area (Pd-Pt/Pd (up to 0.95 cm^2^), Pd-Ag/Pd (up to 0.86 cm^2^), and Pd/Pd (up to 0.81 cm^2^), which was achieved by increasing the number of active adsorption centers due to the unusual morphology of nanoparticles in the composition of catalysts, namely the presence of high-energy high-index facets. Classical spherical Pd_black_/Pd particles in turn had a much smaller electrochemically active surface area of 0.47 cm^2^.

### 3.4. Study of Synthesized Mono- and Bimetallic Nanoparticles Based on Pd, Pd-Ag, Pd-Pt in Hydrogen Transport Processes

**Figure 7:**
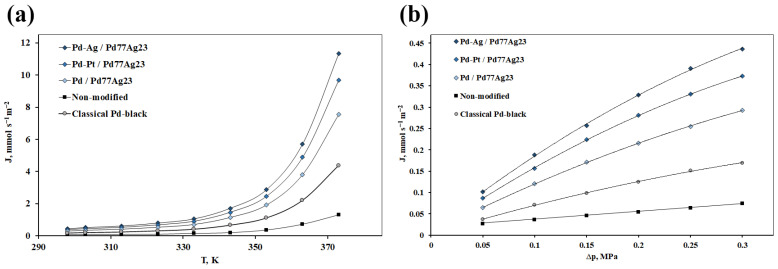
(**a**) Temperature dependence of the hydrogen flux through membranes modified with the developed mono- and bimetallic nanoparticles at gauge pressure of 0.3 MPa; (**b**) Gauge pressure dependence of the hydrogen flux through membranes modified with the developed mono- and bimetallic nanoparticles at a temperature of 25 °C.

The developed mono- and bimetallic catalysts based on Pd were deposited on dense metal Pd_77_Ag_23_ membranes and were investigated in the processes of hydrogen transport. Experiments on the hydrogen permeability of the developed membranes were carried out at low temperatures (20–100 °C) since the greatest effect of a modifying functional layer had been expected in this temperature range. This effect was due to the acceleration of surface processes, which have a predominant effect over bulk processes at sufficiently low temperatures. In the first series of experiments, the temperature dependence of the hydrogen flux for the developed membrane systems was studied. The experiment was from a temperature of 293 K to 373 K with a step of 10 K. Figure 7a shows the corresponding hydrogen fluxes for each studied membrane. It can be seen that the flux values for the modified membranes are several times (up to 8.6 times) higher than the values for the non-modifying Pd_77_Ag_23_ membranes. Probably, the activation of the membrane surface has increased permeability, not only by an increased roughness through applying a modifying layer, but also by a special layer morphology with an increase of active centers number on high-energy facets. Differences in fluxes were also observed between the developed membranes modified with mono- and bimetallic coatings. The Pd-Ag/Pd_77_Ag_23_ membrane had the highest flux of 11.33 mmol s^−1^ m^−2^, the Pd-Pt/Pd_77_Ag_23_ membrane showed a slightly lower value of 9.69 mmol s^−1^ m^−2^, even lower value of 7.56 mmol s^−1^ m^−2^ was shown by Pd/Pd_77_Ag_23_ and the membrane with classical particles Pd_black_/Pd_77_Ag_23_ had the lowest value of 4.37 mmol s^−1^ m^−2^. The demonstrated difference in the hydrogen flux values was due to the synergistic effect of the secondary metal—platinum and silver, which provided an increase in the bimetallic coating activity with respect to hydrogen, as well as the difference in the morphology of nanoparticles synthesized in the composition of the modifying layer, which is caused by the arrangement of atoms of the two different metals on the particle surface as well as by other reasons.

In the second series of experiments, the dependence of the hydrogen flux on the gauge pressure on the inlet membrane side has been considered (Figure 7b). During the experiment, a stepwise pressure increase up to 0.3 MPa was carried out with a step of 0.05 MPa. The studied membrane samples showed the same sequence of values as in the previous experiment: for Pd-Ag/Pd_77_Ag_23_—up to 0.44 mmol s^−1^ m^−2^, for Pd-Pt/Pd_77_Ag_23_—up to 0.37 mmol s^−1^ m^−2^, for Pd/Pd_77_Ag_23_—up to 0.29 mmol s^−1^ m^−2^ and for Pd_black_/Pd_77_Ag_23_—up to 0.17 mmol s^−1^ m^−2^. The obtained values are up to 6.3 times higher than those for the non-coated Pd_77_Ag_23_ membrane, which demonstrates a very unstable and hard-to-record flux under the selected experimental conditions. The curves in Figure 6b clearly show the transition of hydrogen transfer from a surface-limited regime to a diffusion-limited one. The hydrogen flux values for the non-modified membrane are well approximated by the first-order line with the index *n* is 1.0, indicating the desorption-limited permeation regime, which is quite typical for palladium-containing membranes in the low-temperature operation mode. However, the situation changes drastically for modified membranes, and the curves demonstrate a smooth transition to a diffusion-limited mode (the *n* index tends to 0.5). This phenomenon may be due to the obvious acceleration of surface processes, achieved by applying nanoparticles of a controlled pentagonal shape with an increased number of active centers in the modifying membrane surface layer, which have high reactivity with respect to hydrogen.

**Figure 8:**
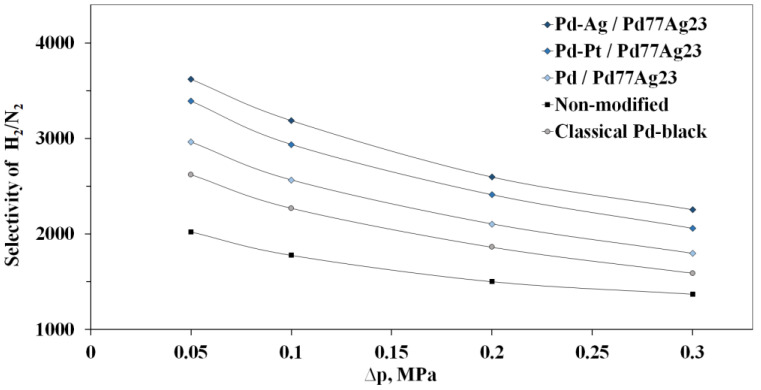
Gauge pressure dependence of selectivity of membranes modified with the developed mono- and bimetallic nanoparticles at a temperature of 25 °C.

The membrane selectivity was determined as the ratio of hydrogen to nitrogen fluxes. The selectivity values were evaluated through gauge pressure dependence of the developed membrane samples (Figure 8). Obviously, the nitrogen flux increased with a pressure increase. Nevertheless, the hydrogen flux increased more, which, in turn, showed sufficiently high selectivity with its slight decrease because of the pressure gradient. Thus, the obtained selectivity values for each of the developed membranes at a pressure of 0.3 MPa were the following: for Pd-Ag/Pd_77_Ag_23_—up to 2254, for Pd-Pt/Pd_77_Ag_23_—up to 2058, for Pd/Pd_77_Ag_23_—up to 1798. The Pd_black_/Pd_77_Ag_23_ membrane modified with classical particles demonstrated a slightly lower selectivity of 1591. The non-coated Pd_77_Ag_23_ membrane had the lowest selectivity of 1264. The difference in selectivities of the developed coated and non-coated membranes was up to 1.6 times. Most probably, a permeate flux significant increase, described in previous experiments on modified membranes’ hydrogen permeability, caused such high selectivity value. No hysteresis was observed with a pressure decrease, which indicates the developed membranes’ mechanical stability to high-pressure gradients.

## 4. Conclusions

A controlled electrochemical synthesis strategy of mono- and bimetallic nanoparticles of a required shape has been developed. The method has made it possible to avoid colloidal synthetic methods because of its simplicity and additional control tools. The fundamental patterns of nanoparticle growth, including the study of the surfactant role and environmental conditions changes (pH) as a control tool for particle shaping, have been investigated with this approach. Thus, a high correspondence between reducing media, created by a low direct current in electrochemical synthesis, and chemical reducing agents in colloidal synthesis have given a mechanistic understanding of synthesis processes and allowed us to obtain nanoparticles with a unique and complex morphology, unreachable by classical methods. The monometallic (palladium), and bimetallic (Pd-Ag and Pd-Pt) nanoparticles with a pentabranched structure were created by the developed synthesis strategy. Such systems are the most promising for the catalysis of surface-sensitive reactions such as the oxidation of alcohol. The developed pentagonally structured mono- and bimetallic nanoparticles have demonstrated a high catalytic activity up to 83.51 mA cm^−2^ and long-term stability in the methanol alkaline oxidation reaction due to the increased electrochemically active surface area achieved by increasing the active centers’ number. The unusual morphology of catalyst nanoparticles, namely, the presence of high-energy high-index facets has allowed increasing the number of active sites. The developed nanoparticles were also studied in the processes of hydrogen transport as a modifying coating for hydrogen-permeable membranes. The membranes demonstrated sufficiently high fluxes up to 11.33 mmol s^−1^ m^−2^ and high selectivity up to 2254. The obvious acceleration of surface processes has been achieved by applying nanoparticles of a given pentagonal shape to the membrane surface, which increased the number of active centers with high reactivity with respect to hydrogen. The reported novel synthesis strategy can potentially be extended to other metal nanoparticle systems. Thus it can provide an effective way to solve long-standing problems in the development of controlled synthetic methods, allowing control of the morphology of nanoparticles and tuning of the required functional properties.

## Data Availability

Not applicable.

## References

[1] Alafnan A., Rizvi S.M.D., Alshammari A.S., Faiyaz S.S.M., Lila A.S.A., Katamesh A.A., Khafagy E.-S., Alotaibi H.F., Ahmed A.B.F. (2022). Gold Nanoparticle-Based Resuscitation of Cefoxitin against Clinical Pathogens: A Nano-Antibiotic Strategy to Overcome Resistance. Nanomaterials.

[2] Jawhari A.H., Hasan N., Radini I.A., Narasimharao K., Malik M.A. (2022). Noble Metals Deposited LaMnO_3_ Nanocomposites for Photocatalytic H_2_ Production. Nanomaterials.

[3] Fernández-Arias M., Vilas A.M., Boutinguiza M., Rodríguez D., Arias-González F., Pou-Álvarez P., Riveiro A., Gil J., Pou J. (2022). Palladium Nanoparticles Synthesized by Laser Ablation in Liquids for Antimicrobial Applications. Nanomaterials.

[4] Song L., Tan K., Ye Y., Zhu B., Zhang S., Huang W. (2022). Amine-Functionalized Natural Halloysite Nanotubes Supported Metallic (Pd, Au, Ag) Nanoparticles and Their Catalytic Performance for Dehydrogenation of Formic Acid. Nanomaterials.

[5] Zhao J., Rafat M.N., Yoon C.-M., Oh W.-C. (2022). Novel Approach to Synthesis of AgZnS and TiO_2_ Decorated on Reduced Graphene Oxide Ternary Nanocomposite for Hydrogen Evolution Effect of Enhanced Synergetic Factors. Nanomaterials.

[6] Fahim T., Laouedj S., Abderrahmane A., Alotaibi S., Younis O., Ali H.M. (2022). Heat Transfer Enhancement in Parabolic through Solar Receiver: A Three-Dimensional Numerical Investigation. Nanomaterials.

[7] Takai T., Shibatani A., Asakuma Y., Saptoro A., Phan C. (2022). Microwave-assisted nanoparticle synthesis enhanced with addition of surfactant. Chem. Eng. Res. Des..

[8] Chan S.S., Low S.S., Chew K.W., Ling T.C., Rinklebe J., Juan J.C., Ng E.P., Show P.L. (2022). Prospects and environmental sustainability of phyconanotechnology: A review on algae-mediated metal nanoparticles synthesis and mechanism. Environ. Res..

[9] Li Y.-Y., Liao H.-G., Rao L., Jiang Y.-X., Huang R., Zhang B.-W., He C.-L., Tian N., Sun S.-G. (2014). Shape Evolution of Platinum Nanocrystals by Electrochemistry. Electrochim. Acta.

[10] Chang Y.-H., Liu C., Rouvimov S., Luo T., Feng S.-P. (2017). Electrochemical Synthesis to Convert a Ag Film into Ag Nanoflowers with High Electrocatalytic Activity. Chem. Commun..

[11] Li A., Chen Y., Zhuo K., Wang C., Wang C., Wang J. (2016). Facile and Shape-Controlled Electrochemical Synthesis of Gold Nanocrystals by Changing Water Contents in Deep Eutectic Solvents and their Electrocatalytic Activity. RSC Adv..

[12] Elrouby M., Abdel-Mawgoud A.M., El-Rahman R.A. (2017). Synthesis of iron oxides nanoparticles with very high saturation magnetization form TEA-Fe(III) complex via electrochemical deposition for supercapacitor applications. J. Mol. Struct..

[13] Chee S.W., Arce-Ramos J.M., Li W., Genest A., Mirsaidov U. (2020). Structural changes in noble metal nanoparticles during CO oxidation and their impact on catalyst activity. Nat. Commun..

[14] Basov A., Dzhimak S., Sokolov M., Malyshko V., Moiseev A., Butina E., Elkina A., Baryshev M. (2022). Changes in Number and Antibacterial Activity of Silver Nanoparticles on the Surface of Suture Materials during Cyclic Freezing. Nanomaterials.

[15] Pan Y.-T., Yang H. (2020). Design of bimetallic catalysts and electrocatalysts through the control of reactive environments. Nano Today.

[16] Lytkina-Payen A., Tabachkova N., Yaroslavtsev A. (2022). Methanol Steam Reforming on Bimetallic Catalysts Based on In and Nb Doped Titania or Zirconia: A Support Effect. Processes.

[17] Maier A., van Oossanen R., van Rhoon G.C., Pignol J.-P., Dugulan I., Denkova A.G., Djanashvili K. (2022). From Structure to Function: Understanding Synthetic Conditions in Relation to Magnetic Properties of Hybrid Pd/Fe-Oxide Nanoparticles. Nanomaterials.

[18] Ruditskiy A., Choi S.-I., Peng H.-C., Xia Y. (2014). Shape-controlled metal nanocrystals for catalytic applications. MRS Bull..

[19] Yang T., Ma Y., Huang Q., Cao G. (2016). Palladium–iridium nanocrystals for enhancement of electrocatalytic activity toward oxygen reduction reaction. Nano Energy.

[20] Tang J., Seo O., Rocabado D.S.R., Koitaya T., Yamamoto S., Nanba Y., Song C., Kim J., Yoshigoe A., Koyama M. (2022). Hydrogen absorption and diffusion behaviors in cube-shaped palladium nanoparticles revealed by ambient-pressure X-ray photoelectron spectroscopy. Appl. Surf. Sci..

[21] Alekseeva S., Strach M., Nilsson S., Fritzsche J., Zhdanov V.P., Langhammer C. (2021). Grain-growth mediated hydrogen sorption kinetics and compensation effect in single Pd nanoparticles. Nat. Commun..

[22] Lao M., Li P., Jiang Y., Pan H., Dou S.X., Sun W. (2022). From fundamentals and theories to heterostructured electrocatalyst design: An in-depth understanding of alkaline hydrogen evolution reaction. Nano Energy.

[23] Ali M.R.K., Wu Y., El-Sayed M.A. (2019). Gold-Nanoparticle-Assisted Plasmonic Photothermal Therapy Advances Toward Clinical Application. J. Phys. Chem. C.

[24] Mansoor A., Khurshid Z., Khan M.T., Mansoor E., Butt F.A., Jamal A., Palma P.J. (2022). Medical and Dental Applications of Titania Nanoparticles: An Overview. Nanomaterials.

[25] Rosales S.A., González F., Moreno F., Gutiérrez Y. (2020). Non-Absorbing Dielectric Materials for Surface-Enhanced Spectroscopies and Chiral Sensing in the UV. Nanomaterials.

[26] Smith A.F., Weiner R.G., Skrabalak S.E. (2016). Symmetry-Dependent Optical Properties of Stellated Nanocrystals. J. Phys. Chem. C.

[27] Yaldagard M., Shahbaz M., Kim H.W., Kim S.S. (2022). Ethanol Electro-Oxidation on Catalysts with S-ZrO_2_-Decorated Graphene as Support in Fuel Cell Applications. Nanomaterials.

[28] Wang S., Yan X., Zhang M., Dong G., Moro R., Ma Y., Ma L. (2022). Real-time size tuning and measuring of silver nanoparticles by cyclic voltammetry and Raman spectroscopy. Mater. Lett..

[29] Filippov S.P., Yaroslavtsev A.B. (2021). Hydrogen energy: Development prospects and materials. Russ. Chem. Rev..

[30] Mironova E.Y., Ermilova M.M., Orekhova N.V., Basov N.L., Yaroslavtsev A.B. (2019). Hydrogen Production by Ethanol Steam Reforming in the Presence of Pd-, Pt-, Ru-, and Ni-Containing Nanodiamonds in Conventional and Membrane Reactors. Membr. Membr. Technol..

[31] Solla-Gullón J., Feliu J.M. (2020). State of the art in the electrochemical characterization of the surface structure of shape-controlled Pt, Au, and Pd nanoparticles. Curr. Opin. Electrochem..

[32] Petriev I.S., Pushankina P.D., Lutsenko I.S., Baryshev M.G. (2021). Anomalous Kinetic Characteristics of Hydrogen Transport through Pd–Cu Membranes Modified by Pentatwinned Flower-Shaped Palladium Nanocrystallites with High-Index Facets. Tech. Phys. Lett..

[33] Petriev I.S., Pushankina P.D., Lutsenko I.S., Baryshev M.G. (2021). The Influence of a Crystallographically Atypical Pentagonal Nanostructured Coating on the Limiting Stage of Low-Temperature Hydrogen Transport through Pd–Cu Membranes. Dokl. Phys..

[34] Vidal-Iglesias F.J., Solla-Gullón J., Herrero E., Montiel V., Aldaz A., Feliu J.M. (2011). Evaluating the ozone cleaning treatment in shape-controlled Pt nanoparticles: Evidences of atomic surface disordering. Electrochem. Commun..

[35] Petriev I., Pushankina P., Lutsenko I., Shostak N., Baryshev M. (2020). Synthesis, Electrocatalytic and Gas Transport Characteristics of Pentagonally Structured Star-Shaped Nanocrystallites of Pd-Ag. Nanomaterials.

[36] Petriev I., Pushankina P., Shostak N., Baryshev M. (2022). Gas-Transport Characteristics of PdCu–Nb–PdCu Membranes Modified with Nanostructured Palladium Coating. Int. J. Mol. Sci..

[37] Petriev I.S., Baryshev M.G., Voronin K.A., Lutsenko I.S., Pushankina P.D., Kopytov G.F. (2020). Gas Transmission Properties of Pd–Ag Membranes Coated with Modifying Layer. Russ. Phys. J..

[38] Garnier E., Vidal-Iglesias F.J., Feliu J.M., Solla-Gullón J. (2019). Surface Structure Characterization of Shape and Size Controlled Pd Nanoparticles by Cu UPD: A Quantitative Approach. Front. Chem..

[39] Lv H., Xu D., Sun L., Henzie J., Suib S.L., Yamauchi Y., Liu B. (2019). Ternary Palladium–Boron–Phosphorus Alloy Mesoporous Nanospheres for Highly Efficient Electrocatalysis. ACS Nano.

[40] Strasser P., Gliech M., Kuehl S., Moeller T. (2018). Electrochemical processes on solid shaped nanoparticles with defined facets. Chem. Soc. Rev..

[41] Petriev I., Pushankina P., Bolotin S., Lutsenko I., Kukueva E., Baryshev M. (2021). The influence of modifying nanoflower and nanostar type Pd coatings on low temperature hydrogen permeability through Pd-containing membranes. J. Membr. Sci..

[42] King M.E., Personick M.L. (2017). Defects by design: Synthesis of palladium nanoparticles with extended twin defects and corrugated surfaces. Nanoscale.

[43] Ruiz-Ruiz V.-F., González-Olvera R., Díaz-Pardo R., Betancourt I., Zumeta-Dubé I., Díaz D., Farfán N., Arellano-Jiménez M.J. (2018). Mechanochemically obtained Pd–Ag nanoalloys. Structural considerations and catalytic activity. Materialia.

[44] Vegard L. (1921). Die Konstitution der Mischkristalle und die Raumfüllung der Atome. Z. Phys..

[45] Bochicchio D., Ferrando R., Novakovic R., Panizon E., Rossi G. (2014). Chemical ordering in magic-size Ag–Pd nanoparticles. Phys. Chem. Chem. Phys..

[46] Guo M., He H., Zhang Z., Liu Z., Xie F., Shan B., Duan X. (2020). Structural optimization and melting behavior investigation of Pd-Ag bimetallic nanoparticles by molecular simulations. Comput. Mater. Sci..

[47] Paz-Borbón L.O., Johnston R.L., Barcaro G., Fortunelli A. (2008). Structural motifs, mixing, and segregation effects in 38-atom binary clusters. J. Chem. Phys..

[48] Yin Z., Lin L.L., Ma D. (2014). Construction of Pd-based nanocatalysts for fuel cells: Opportunities and challenges. Catal. Sci. Technol..

[49] Wang H., Sheng L., Zhao X., An K., Ou Z., Fang Y. (2018). One-step synthesis of Pt-Pd catalyst nanoparticles supported on few-layer graphene for methanol oxidation. Curr. Appl. Phys..

[50] Hanifah M.F.R., Jaafar J., Othman M.H.D., Ismail A.F., Rahman M.A., Yusof N., Aziz F., Rahman N.A.A. (2019). One-pot synthesis of efficient reduced graphene oxide supported binary Pt-Pd alloy nanoparticles as superior electro-catalyst and its electro-catalytic performance toward methanol electro-oxidation reaction in direct methanol fuel cell. J. Alloys Compd..

[51] Zhang W., Yao Q., Wu X., Fu Y., Deng K., Wang X. (2016). Intimately coupled hybrid of graphitic carbon nitride nanoflakelets with reduced graphene oxide for supporting Pd nanoparticles: A stable nanocatalyst with high catalytic activity towards formic acid and methanol electrooxidation. Electrochim. Acta.

